# Locking nail versus locking plate for proximal humeral fracture fixation in an elderly population: a prospective randomised controlled trial

**DOI:** 10.1186/s12891-019-2399-1

**Published:** 2019-01-10

**Authors:** Johannes E. Plath, Christian Kerschbaum, Tobias Seebauer, Rainer Holz, Daniel J. H. Henderson, Stefan Förch, Edgar Mayr

**Affiliations:** 1 Department of Trauma, Orthopaedic, Plastic and Hand Surgery, University Hospital of Augsburg, Stenglinstrasse 2, 86156 Augsburg, Germany; 2 Department of Trauma and Orthopaedics, Leeds General Infirmary, Leeds, UK

**Keywords:** Proximal humeral fracture, Geriatric traumatology, Locking proximal humeral nail, Locking proximal humeral plate

## Abstract

**Background:**

Proximal humeral fractures (PHFs) are the third most common fracture in older patients. The purpose of the study was to prospectively evaluate the outcomes of PHF fixation with a locking blade nail (LBN) or locking plate (PHILOS) osteosynthesis in a homogeneous elderly patient population.

**Methods:**

Inclusion criteria were an age > 60 years and the capacity to give informed consent. Patients with isolated tuberosity fractures, previous trauma or surgery, advanced osteoarthritis, fracture dislocation, pathological fractures, open fractures, neurological disorders, full-thickness rotator cuff tears, fracture line at the nail entry point or severely reduced bone quality intra-operatively were excluded.

Eighty one patients with PHFs were randomised to treatment using LBN or PHILOS. Outcome measures comprised Constant score, age and gender adjusted Constant score, DASH score, VAS for pain, subjective overall condition of the shoulder (1–6) and active shoulder range-of-motion in flexion and abduction. Plain radiographs were obtained in two planes. All data were collected by an independent observer at 3, 6 and 12 months postoperatively.

**Results:**

Thirteen patients were excluded intra-operatively due to rotator cuff tears, fracture morphology or poor bone-quality. Of the remaining 68 patients, 27 in the LBN and 28 in the PHILOS group completed the full follow-up. Mean age at surgery was 75.6 years and the majority of PHFs were three-part fractures (49 patients). Baseline demographics between groups were comparable.

All outcome measures improved between assessments (*p* < 0.001). The LBN group showed improved DASH scores as compared to PHILOS at 12 months (*p* = 0.042) with fewer incidences of secondary loss of reduction and screw cut-out (*p* = 0.039). A total of 29 complications (in 23 patients) were recorded, 13 complications (in 12 patients) in the LBN group and 16 complications (in 11 patients) in the PHILOS group (*p* = 0.941). No significant inter-group difference was observed for any other outcome measures, nor was fracture morphology seen to be associated with clinical outcome or complication rate.

**Conclusions:**

At short-term follow-up, LBN osteosynthesis yielded similar outcomes and complication rates to PHILOS plate fracture fixation in an elderly patient population, though with a significantly lower rate of secondary loss of reduction and screw cut-out.

**Registration trial:**

No. DRKS00015245 at Deutsches Register Klinischer Studien, registered: 22.08.2018, retrospectively registered.

## Background

Proximal humeral fractures (PHFs) are the third most common fracture in geriatric patients, typically associated with systemic osteoporosis, and its’ incidence is expected to triple over the coming three decades [1, 2]. Numerous surgical techniques for the treatment of PHFs have been described and developed, with locking plate osteosynthesis often considered the “gold standard” [3, 4]. However, a high rate of complications, especially varus displacement with screw cut-out, has been widely reported with this technique, and subsequently osteoporosis, patient age and insufficient medial cortical support are generally considered the main risk factors for failure of PHF fixation [3, 5–14].

Humeral nails have undergone significant evolution and innovation over the past 40 years, & where proximal humeral nailing was previously regarded as a technique appropriate only for simple surgical neck fractures, modern locking nail designs have been developed that allow treatment of more complex PHFs [15–17].

In a geriatric population, locking nail osteosynthesis provides a number of potential advantages over plating, including higher primary stability, a high initial load bearing capacity and a minimally invasive surgical approach [4, 15, 16, 18–20].

Although 70% of PHFs have been shown to occur in patients aged 60 years and over, to our knowledge, no study to date has compared the outcomes of locking plate osteosynthesis and locking nail osteosynthesis in a homogeneous elderly population [2].

A number of studies report similar clinical outcomes of these two procedures, however, to date, only two published studies in the literature present the results of prospective randomised and controlled studies, and only one of them includes complex PHFs [4, 16, 20–27].

The aim of the present study, therefore, was to prospectively evaluate the outcomes of elderly patients with PHFs who were treated with either a locking plate osteosynthesis or locking nail osteosynthesis, in a randomised controlled study design.

It was hypothesised that locking nail osteosynthesis, using a modern locking nail design, provides at least equivalent clinical outcomes, with less secondary fracture displacement, as compared to the “gold standard” of locking plate osteosynthesis.

## Methods

### Patient selection

This was a single centre randomised controlled trial, with randomisation performed using a random numbers list. The study was performed according to the CONSORT guidelines.

One trauma surgeon, who was not directly involved in the surgical intervention enrolled the patients and evaluated the outcome mesurements. All surgery was performed by one of three experienced trauma surgeons.

The aim of the present study was to prospectively evaluate the outcomes of elderly patients with PHFs who were treated with either a locking plate osteosynthesis or locking nail osteosynthesis, in a randomised controlled study design.

The study was performed in accordance with the Declaration of Helsinki and the study protocol was approved by the local ethics committee before initiation of this study (approval No. 442–13). All patients provided written informed consent to participation.

Between September 2012 and February 2015, all patients presenting to our institution, a level I trauma center, with an acute isolated PHF were prospectively enrolled. Further inclusion criteria were an age of 60 years or older and the capacity to provide informed consent.

Exclusion criteria were isolated tuberosity fractures, previous trauma or surgery of the affected shoulder, advanced osteoarthritis, fracture dislocation, pathological fractures, open fractures, neurological disorders, full-thickness rotator cuff tears as well as intra-operative change of treatment due to a fracture line through the nail entry point or where bone quality was considered not amenable to stable fixation with either implant.

81 patients met the inclusion criteria and were assigned to treatment groups. Intraoperatively, 13 patients were excluded from the study. Seven patients showed a full-thickness rotator cuff tear and 4 patients scheduled for osteosynthesis were primarily converted to a reverse total shoulder replacement due to severely reduced bone quality. In 2 patients assigned to the LBN group the fracture was found to be running through the nail entry point at the humeral head apex making a stable nail osteosynthesis impossible. These patients were treated with a PHILOS plate and excluded from the study.

**Fig. 1:**
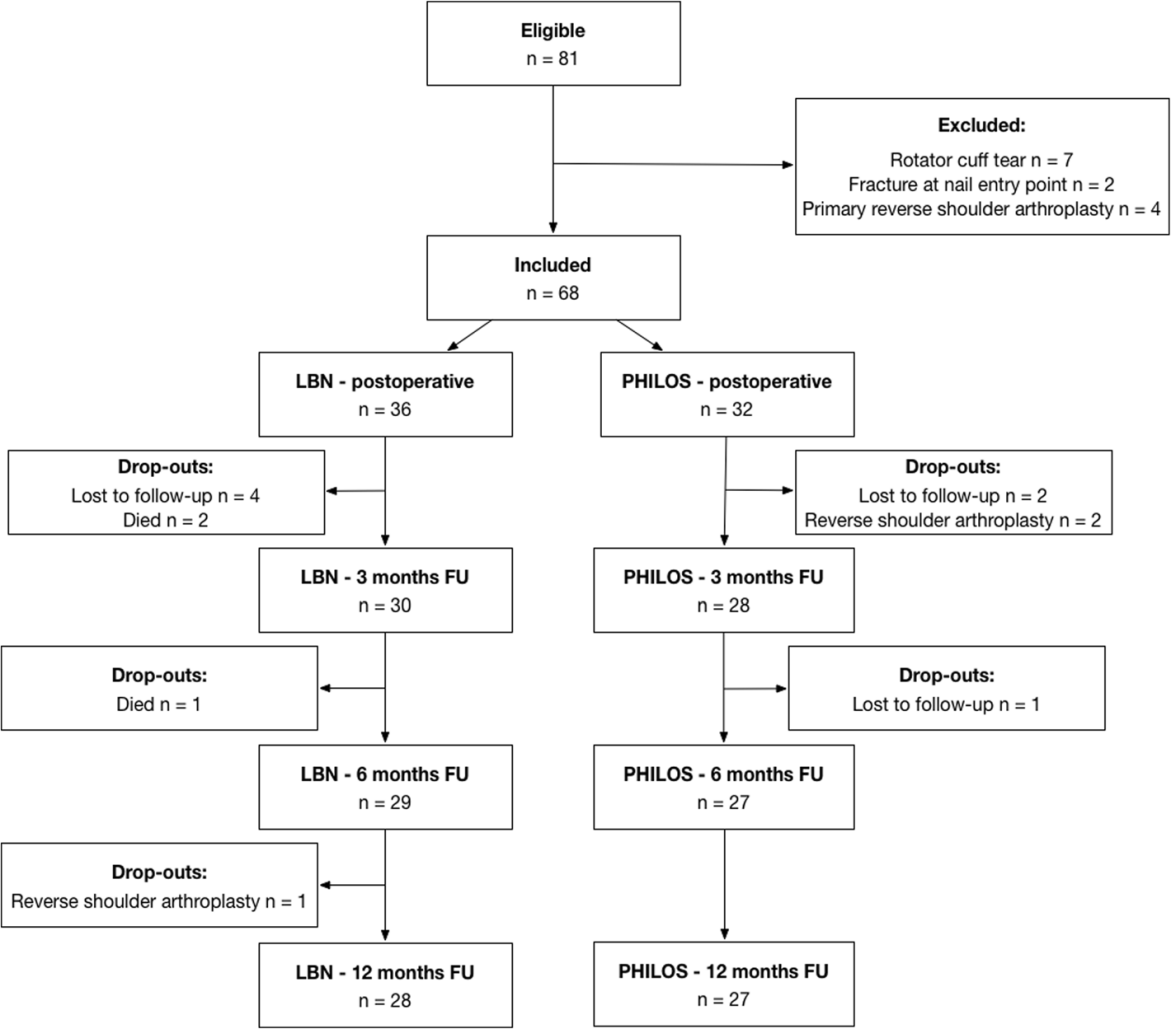
Flow diagram of patient enrolment and analysis n – number, FU – follow-up

Of the remaining 68 patients, 13 patients were lost to follow-up, for various reasons. Three patients in the LBN group died from causes unrelated to the surgery before completing the 12-months follow-up. In total, 28 patients in the LBN group and 27 patients in the PHILOS group completed follow-up. Further details of patient enrolment and analysis are provided in the study’s flow diagram (Fig. 1).

### Pre-operative evaluation

True antero-posterior and lateral radiographs were obtained in all patients. An additional CT-scan was performed only if required for surgical planning. Fractures were classified according to the Neer [28] and Arbeitsgemeinschaft für Osteosynthesefragen (AO) classifications [29].

### Surgical intervention

Surgery was performed with patients in the beach-chair position under general anaesthesia.

#### Nail group

**Fig. 2:**
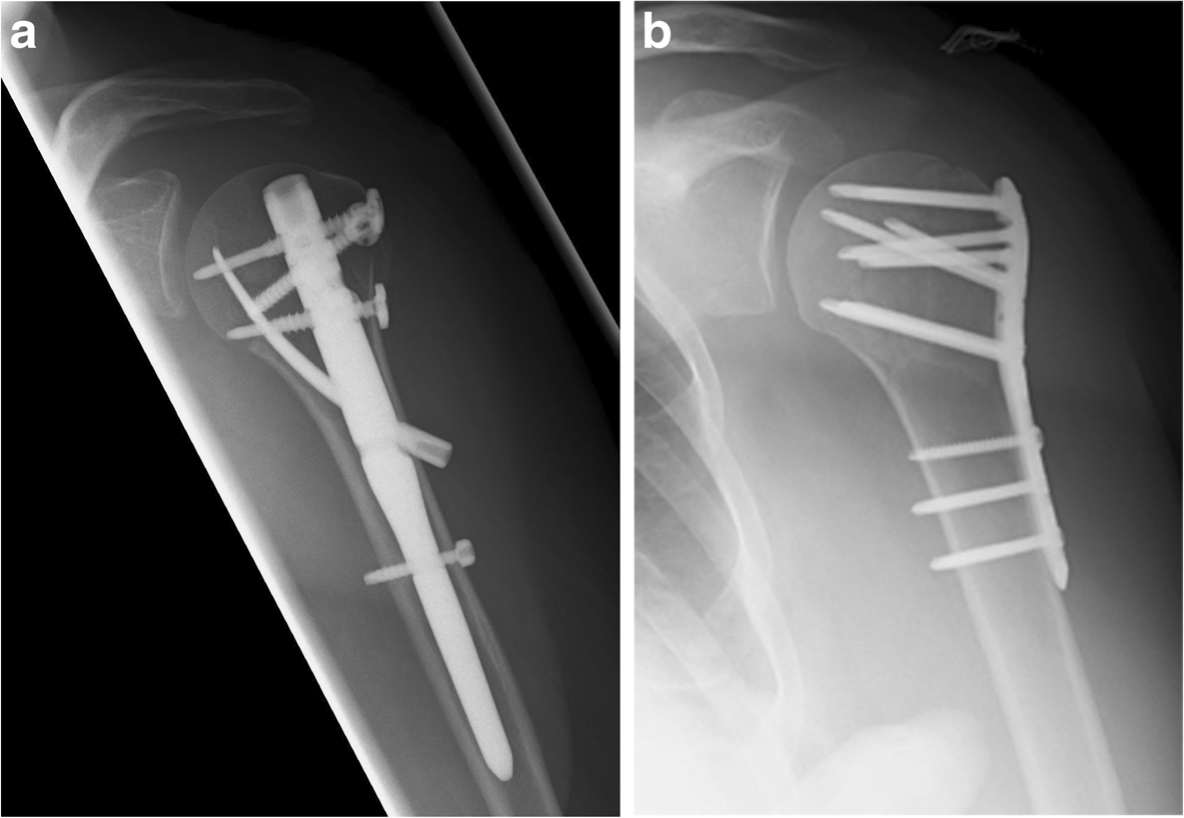
**a**/**b** Post-operative images of Locking Blade Nail, LBN (**a**) and Proximal Humerus InterLocking System, PHILOS (**b**) osteosynthesis

The nail used in the current study is a straight antegrade locking nail that was specially designed to treat more complex osteoporotic PHFs and features a distally inserted locking blade (Locking Blade Nail, LBN, Marquard Medizintechnik Europe) [17]. Four cross-locking cancellous screws are used for proximal fixation; one each for greater and lesser tuberosity fixation and two lateral screws that additionally lock the blade. This creates a triangular construct which aims to add medial calcar support and to increase implant purchase in the metaphyseal region of the humeral head. Mobile washers provide additional anchorage for suture fixation. (Fig. 2 a/b).

The nail is inserted via a 4–5 cm anterolateral transdeltoid approach following fracture reduction under image-intensifier guidance. The supraspinatus tendon is split in the line of its fibres and a guide wire is introduced into the nail entry point at the apex of the humeral head. The medullary canal is opened using a cannulated drill, and the nail inserted over the guide-wire. Proximal and distal locking is achieved by use of an attached guide. Optional non-absorbable tension band sutures (FibreWire, Arthrex Inc., Naples, USA) may then be placed through the rotator cuff and secured to washers on the proximal screws. Finally, the rotator cuff tendon and deltoid are repaired and the skin closed in layers finishing with a non-absorbable continuous suture.

#### Plate group

Locking plate osteosynthesis was performed using the PHILOS locking plate system (Proximal Humerus InterLocking System, DePuy Synthes, Solothurn, Switzerland) via a deltopectoral or lateral transdeltoid approach. Fracture reduction was obtained in a closed fashion under image-intensifier whenever possible. In three- and four-part fractures, non-absorbable sutures (FibreWire, Arthrex Inc., Naples, USA) were used to secure the greater and/or lesser tuberosities to the plate. A minimum of 3 screws were used at the humeral shaft (2 locking and 1 non-locking screw) and a minimum of 6 locking screws, including 2 calcar support screws, to fix the plate proximally.

### Post-operative treatment

In both groups, active and passive range-of-motion exercises were initiated on the day following surgery, as pain allowed, without restriction. No immobilization was used in either group. Patients were instructed not to load-bear with the affected shoulder for 6 weeks postoperatively wherever possible.

### Post-operative evaluation

Clinical follow-up was performed by a single independent observer (a board certified orthopaedic trauma surgeon) at 3, 6 and 12 months postoperatively. Radiographic images were obtained postoperatively and at each follow-up time-point.

#### Evaluation of shoulder function

Constant Murley [30] of the affected side were recorded as primary outcome measure to evaluate shoulder function. Age and gender adjusted Constant Murley score results were calculated according to Katolik et al. [31].

Secondary outcome measures included Disabilities of the Arm, Shoulder and Hand (DASH) scores, Visual Analog Scale (VAS) score for the current pain level (0 representing no pain and 10 representing maximal imaginable pain), a subjective grade for the overall condition of the shoulder within the past month (excellent, good, satisfactory, sufficient, not sufficient, poor) and active shoulder flexion and abduction range-of-motion [32].

#### Radiographic evaluation

Plain radiographs were obtained in two planes and assessed for postoperative implant position, tuberosity resorption and secondary superior migration of the humeral head, osteonecrosis as well as secondary failure of fixation.

Implant malposition was defined as a sub-optimal implant position on postoperative imaging without mechanical consequences for shoulder function. Secondary failure of fixation was defined as a significant loss of reduction occurring over the course of follow-up. All radiographs were analysed by the same independent observer conducting follow-up.

### Sample size calculation

Sample size calculation was based on a mean difference of 10% between groups of the age and gender adjusted Constant Murley score outcomes [31]. With a standard deviation of 10 points, an alpha of 5% and a power of 80%, a sample size of 25 patients in each study group was required. To compensate potential drop-outs during follow-up a minimum of 32 patients per group were included.

### Statistical analysis

Statistical analyses were performed using StatsDirect (StatsDirect Ltd., Cheshire, UK). Quantitative data comparison between groups was analysed using the Mann-Whitney-U-Test. The Friedmann test was used for paired non-normally distributed data. Dichotomous data were computed by the Fisher-Freeman-Halton test. The level of significance was set at *p* < 0.05.

## Results

Of the final 68 patients, mean age at surgery was 75.6 years (60–92). The majority of cases were three-part fractures (49 patients) according to the Neer classification and AO 11-B1 fractures (30 patients) according to the AO classification.

No differences between the LBN and the PHILOS groups were seen with regard to patient number, age, side, involvement of the dominant shoulder, the American Society of Anaesthesiologists (ASA) physical status classification or fracture morphology (Table 1).

**Table 1:** Patient characteristics according to treatment group*.* AO- Arbeitsgemeinschaft für Osteosynthesefragen, ASA – American Society of Anaesthesiologists, y - years

		Overall	LBN	PHILOS	*P* value
No. of patients (male / female)		68 (17 / 51)	36 (10 / 26)	32 (7 / 25)	0.780
Mean age at surgery (range), y		75.6 (60–92)	71.1 (60–87)	77.1 (60–92)	0.094
Right / Left ratio (%)		27 / 41 (40 / 60)	14 / 22 (39 / 61)	13 / 19 (41 / 59)	> 0.999
Dominant side affected (%)		29 / 39 (43 / 57)	15 / 21 (42 / 58)	14 / 18 (44 / 56)	> 0.999
ASA physical status classification	1	17 (25)	6 (17)	11 (34)	0.174
	2	27 (40)	16 (44)	11 (34)	
	3	23 (34)	13 (36)	10 (31)	
	4	1 (2)	1 (3)	0 (0)	
Neer Classification (%)	2-part	9 (13)	5 (14)	4 (13)	0.404
	3-part	49 (72)	25 (69)	24 (75)	
	4-part	10 (15)	6 (17)	4 (13)	
AO Classification (%)	A2	6 (9)	3 (8)	3 (9)	0.233
	A3	7 (10)	6 (17)	1 (3)	
	B1	30 (44)	16 (44)	14 (44)	
	B2	2 (3)	2 (6)	0 (0)	
	C1	17 (25)	6 (17)	11 (34)	
	C2	6 (9)	3 (8)	3 (9)	

Mean duration of surgery was 51.9 min ± 10.3 (range 34–91) in the LBN group and 57.8 min ± 18.2 (range 30–114) in the PHILOS group (*p* = 0.226). Neither the mean intraoperative image intensifier time nor the mean length of postoperative hospital stay was seen to differ between treatment groups (*p* = 273 and 0.686) (Table 2).

**Table 2:** Duration of surgery, exposure to radiation and length of in-hospital stay according to treatment group d – days, min – minutes, sec - seconds

	Overall	LBN	PHILOS	*P* value
Mean duration of surgery ± SD (range), min	54.7 ± 14.8 (30–114)	51.9 ± 10.3 (34–91)	57.8 ± 18.2 (30–114)	0.226
Mean intraoperative image intensifier time ± SD (range), sec	43.8 ± 25.7 (6–116)	43.5 ± 18.7 (12–78)	44.2 ± 32.2 (6–116)	0.273
Mean length of postoperative in-hospital stay ± SD (range), d	7.3 ± 3.4 (3–23)	7.4 ± 3.1 (3–15)	7.3 ± 3.8 (3–23)	0.686

Follow-up was conducted at a mean of 3.0 months, 5.9 months and 12.8 months postoperatively.

**Fig. 3:**
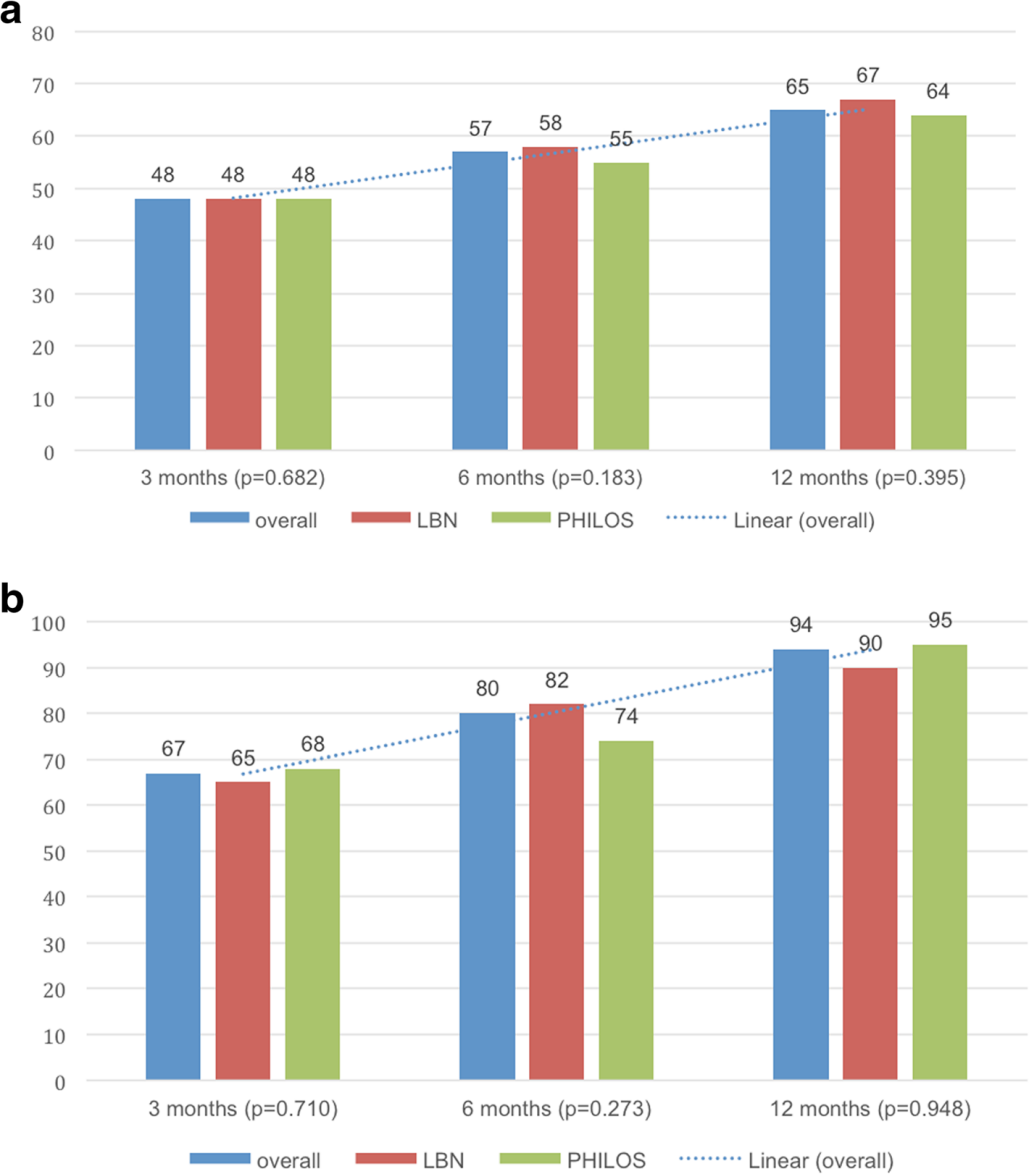
**a**/**b**: Median Constant Murley [30] score (**a**) and age/gender adjusted Constant Murley [31] score (**b**) outcomes: overall, in the LBN group and in the PHILOS group at 3, 6 and 12 months.

Median Constant scores (age and gender adjusted) improved from 48 points (67%) at 3 months to 57 points (80%) at 6 months and 65 points (94%) at 12 months (*p* < 0.001). (Fig. 3a/b) There were no significant differences seen between groups at any follow-up point (Table 3).

**Table 3:** Median Constant Murley [30] score and mean age/gender adjusted Constant Murley [31] score outcomes, Disabilities of the Arm, Shoulder and Hand (DASH) score [32], Visual Analog Scale (VAS) score for pain level, subjective grade for the overall shoulder condition within the past month (1-excellent, 2-good, 3-satisfactory, 4-sufficient, 5-not sufficient, 6-poor) and mean shoulder flexion and abduction range of motion: overall, in the LBN group and in the PHILOS group at 3, 6 and 12 months

		Overall	LBN	PHILOS	*P* value
Constant score ± SD (range)	at 3 months	48 ± 15.2 (10–79)	48 ± 15.0 (13–73)	48 ± 16.0 (10–79)	0.587
	at 6 months	57 ± 15.4 (15–87)	58 ± 15.0 (15–83)	55 ± 16.0 (19–87)	0.119
	at 12 months	65 ± 20.0 (15–93)	67 ± 20.2 (15–93)	64 ± 20.2 (21–93)	0.659
Constant score (age and gender adjusted) ± SD (range)	at 3 months	67.0 ± 21.4 (14–100)	65.3 ± 21.1 (19–100)	68.0 ± 21.9 (14–100)	0.637
	at 6 months	80.4 ± 24.0 (4–100)	82.4 ± 21.1 (22–100)	74.0 ± 26.3 (4–100)	0.203
	at 12 months	93.6 ± 27.5 (22–100)	90.0 ± 27.6 (22–100)	95.0 ± 27.8 (32–100)	0.917
DASH score ± SD (range)	at 3 months	51 ± 17.3 (29–93)	51 ± 17.0 (32–89)	52 ± 17.8 (29–93)	0.505
	at 6 months	48 ± 15.6 (26–89)	41 ± 14.8 (27–85)	45 ± 16.0 (26–89)	0.110
	at 12 months	45 ± 18.5 (22–85)	34 ± 17.8 (22–85)	42 ± 19.1 (24–84)	0.042
VAS pain (1–10) ± SD (range)	at 3 months	4 ± 1.9 (0–8)	3 ± 2.0 (0–7)	4 ± 1.9 (0–8)	0.318
	at 6 months	3 ± 1.5 (0–5)	2 ± 1.6 (0–5)	3 ± 1.3 (0–5)	0.186
	at 12 months	0 ± 1.7 (0–5)	0 ± 1.8 (0–5)	1 ± 1.6 (0–5)	0.766
subjective condition (1–6) ± SD (range)	at 3 months	4 ± 1.2 (1–6)	3 ± 1.3 (1–6)	4 ± 1.1 (1–5)	0.459
	at 6 months	3 ± 1.2 (1–6)	3 ± 1.3 (1–6)	3 ± 1.1 (1–5)	0.176
	at 12 months	2 ± 1.1 (1–5)	2 ± 1.3 (1–5)	2 ± 0.8 (1–4)	0.751
Flexion ± SD (range), degree	at 3 months	85.5 ± 33.9 (10–170)	88.3 ± 30.2 (10–150)	82.9 ± 37.4 (30–170)	0.356
	at 6 months	110.8 ± 38.0 (20–180)	119.6 ± 44.5 (20–180)	101.9 ± 28.6 (50–180)	0.150
	at 12 months	127.0 ± 44.2 (20–180)	124.1 ± 45.4 (20–180)	130.0 ± 43.5 (60–180)	0.694
Abduction ± SD (range), degree	at 3 months	80.2 ± 31.2 (30–170)	83.7 ± 26.8 (30–150)	77.0 ± 37.4 (30–170)	0.228
	at 6 months	101.5 ± 40.8 (20–180)	110.4 ± 41.7 (35–180)	92.7 ± 38.6 (20–180)	0.156
	at 12 months	122.1 ± 49.1 (30–180)	120.9 ± 46.9 (30–180)	123.3 ± 52.0 (30–180)	0.661

**Fig. 4:**
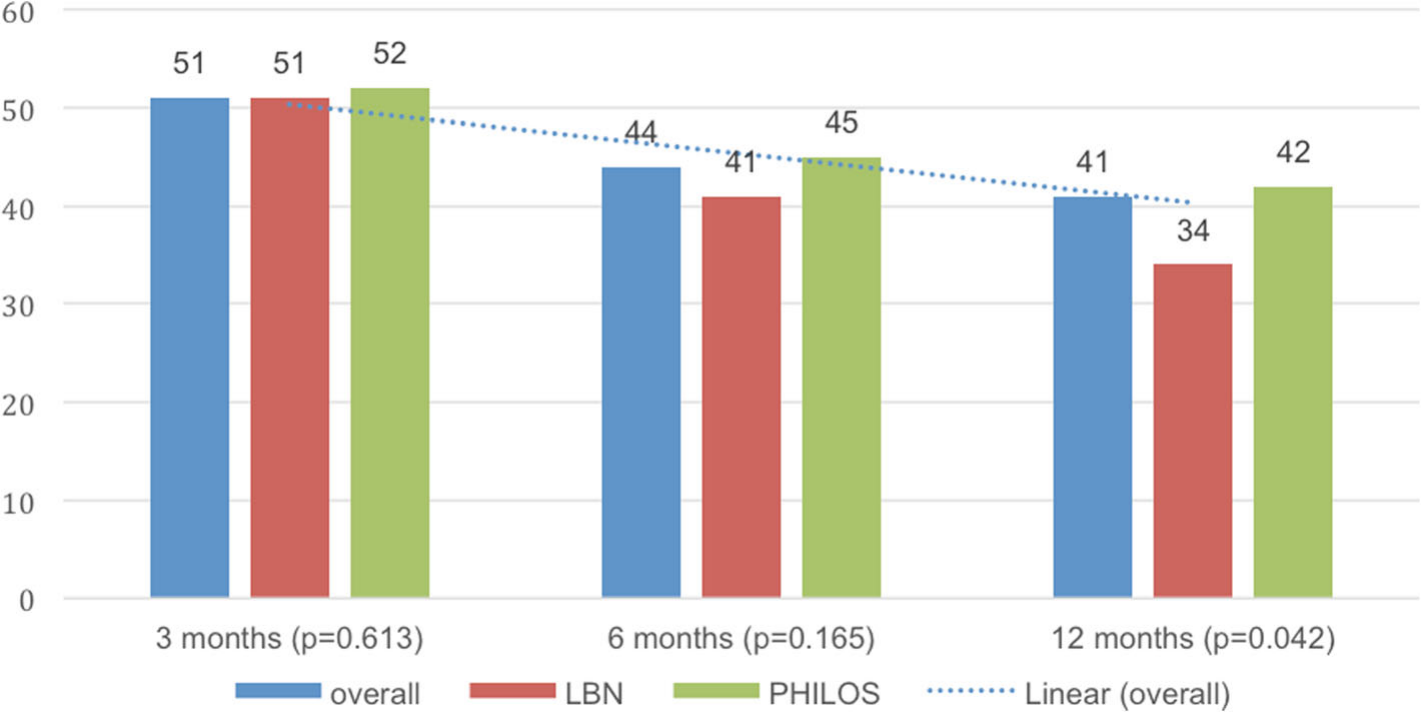
Median Disabilities of the Arm, Shoulder and Hand (DASH) score [32] outcomes: overall, in the LBN group and in the PHILOS group at 3, 6 and 12 months

DASH scores were seen to decrease from a median of 51 points at 3 months to 44 points at 6 months and to 41 points at 12 months (*p* < 0.001) (Fig. 4). While no inter-group differences could be detected at 3 and 6 months postoperatively, the LBN group showed a reduced DASH score at 12 months as compared to the PHILOS group (*p* = 0.042) (Table 3).

**Fig. 5:**
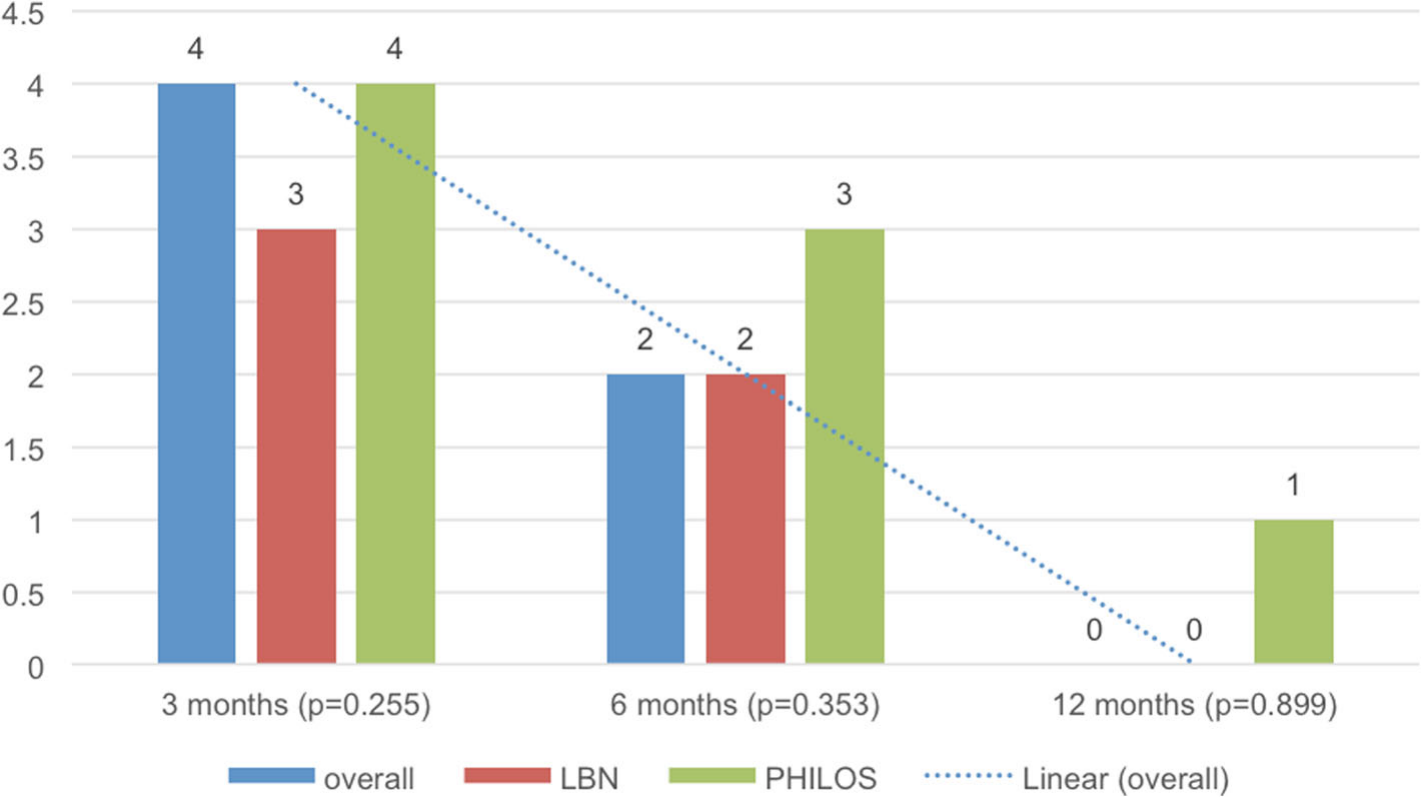
Median Visual Analog Scale (VAS) score for pain level: overall, in the LBN group and in the PHILOS group at 3, 6 and 12 months

The median VAS level for pain also decreased significantly during the course of follow-up from 4 at 3 months to 2 at 6 months and to 0 at 12 months (*p* < 0.001) with no significant inter-group difference at any follow-up time-point (Fig. 5) (Table 3).

**Fig. 6:**
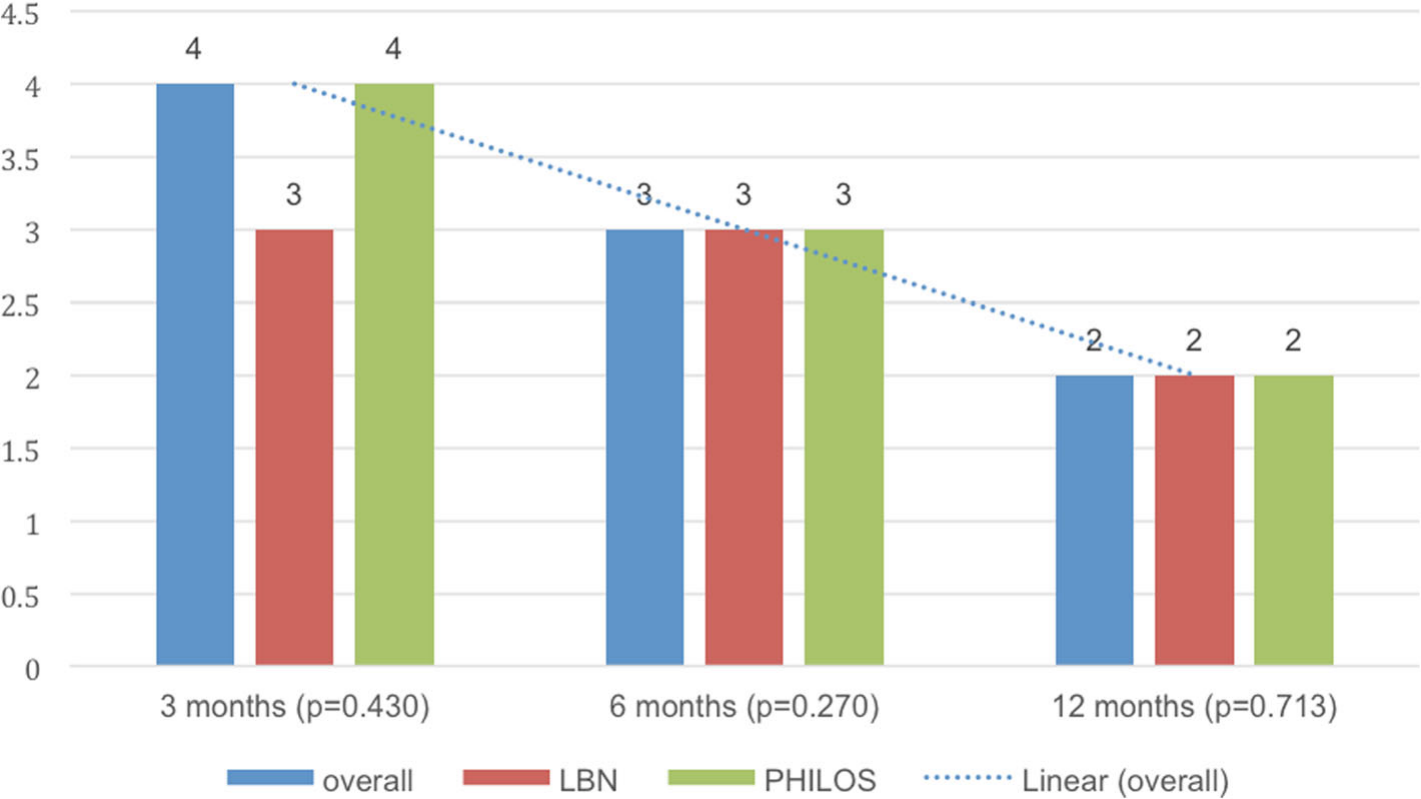
Median subjective grade for the overall condition of the shoulder (1-excellent, 2-good, 3- satisfactory, 4-sufficient, 5-not sufficient, 6-poor): overall, in the LBN group and in the PHILOS group at 3, 6 and 12 months.

On average, patients rated the overall condition of their shoulder as “sufficient” at 3 months, “satisfactory” at 6 months and “good” at 12 months (*p* < 0.001) (Fig. 6). No significant differences between the LBN and PHILOS group were detected (Table 3).

Shoulder range of flexion and abduction improved significantly between all follow-up time points (*p* < 0.001 and *p* < 0.001). There was no significant inter-group difference at any time point (Table 3).

Fracture morphology and complexity was not seen to correlate with any outcome measure, in either the LBN or PHILOS group, at final follow-up (*p* >  0.05).

A total of 29 complications (in 23 patients) were recorded, 13 complications (in 12 patients) in the LBN group and 16 complications (in 11 patients) in the PHILOS group (*p* = 0.941) (Table 4).

**Table 4:** Complications and reoperations: overall, in the LBN group and in the PHILOS group w/o – without

		Overall	LBN	PHILOS	*P* value
Number of patients with complication (%)		23 (33.8)	12 (33.3)	11 (34.3)	> 0.999
Complications (%)	Malposition of impants	6 (8.8)	4 (11.1)	2 (6.3)	0.676
	Loss of reduction of humeral head with screw cut-out	10 (14.7)	2 (5.6)	8 (25.0)	0.039
	Loss of reduction of humeral head w/o screw cut-out	2 (2.9)	2 (5.6)	0 (0)	0.494
	Loss of reduction of greater tuberosity	1 (1.5)	0 (0)	1 (3.1)	> 0.999
	Tuberosity resorption / head migration	5 (7.4)	3 (8.3)	2 (6.3)	> 0.999
	Migration without tuberosity resorption	1 (1.5)	1 (2.8)	0 (0)	> 0.999
	Osteonecrosis of humeral head	2 (2.9)	1 (2.8)	1 (3.1)	> 0.999
	Axillary nerve lesion	1 (1.5)	0 (0)	1 (3.1)	> 0.999
	Adhesive capsulitis	1 (1.5)	0 (0)	1 (3.1)	> 0.999
Total number of complications		29	13	16	0.941
Revision surgery indicated (%)		14 (20.6)	5 (13.9)	9 (28.1)	0.229
Revisions performed (%)	Reverse shoulder arthroplasty	3 (4.4)	1 (2.8)	2 (6.3)	0.598
	Arthrolysis and implant removal	9 (13.2)	4 (11.1)	5 (15.6)	0.725
Total number of performed revisions		12 (17.6)	5 (13.9)	7 (21.9)	0.527

**Fig. 7:**
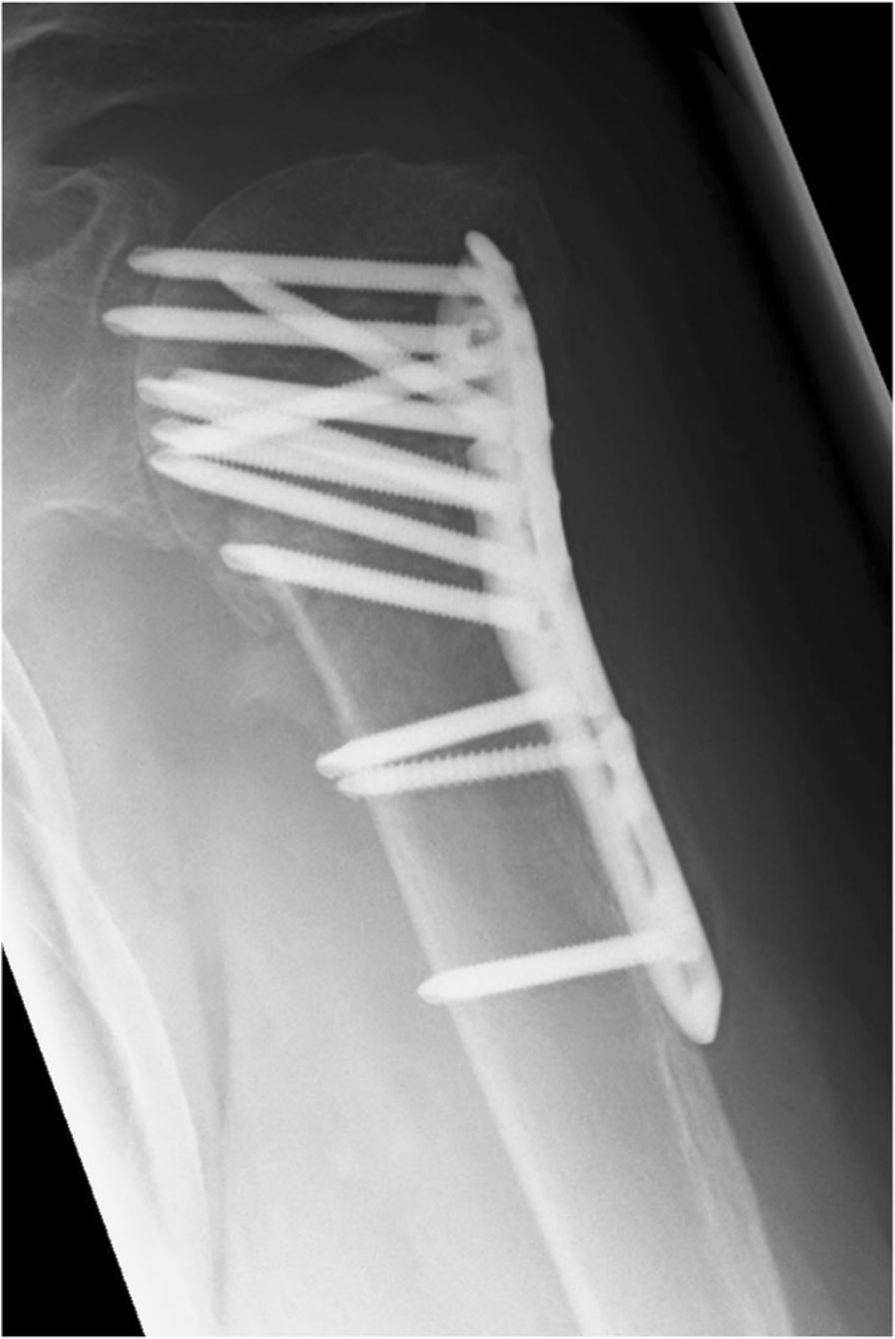
Secondary varus displacement of the humeral head with screw cut-out following PHILOS plate osteosynthesis

The most frequent complication was a secondary varus displacement of the humeral head with screw cut-out, which occurred in 2 patients in the LBN group and 10 patients in the PHILOS group, demonstrating a significant inter-group difference (*p* = 0.039) (Fig. 7). A secondary failure of fixation, without screw cut-out, was noted in 2 patients in the LBN group (*p* = 0.494).

The second most frequent complication was malpositioning of the implant on post-operative imaging, which was seen in 4 patients in the LBN and 2 patients in the PHILOS cohort (*p* = 0.676). In none of these cases was the sub-optimal implant positioning considered to represent a potential mechanical issue for shoulder function and therefore revision was not indicated in any case.

Tuberosity resorption and secondary humeral head migration occurred in 5 patients during the course of follow-up, 3 patients with LBN and 2 patients with PHILOS osteosynthesis (*p* >  0.999).

Further complications were humeral head migration without tuberosity resorption (*n* = 1), loss of reduction of the greater tuberosity (n = 1), humeral head osteonecrosis (*n* = 2), axillary nerve lesion (*n* = 1) and adhesive capsulitis (*n* = 1). No patient suffered a postoperative infection.

Overall, re-operation was indicated in 14 patients (5 LBN and 9 PHILOS) for one or more complications. In 12 patients further surgery was performed during the follow-up period, with implant removal and arthrolysis being the most frequent secondary procedure (*n* = 9). In 3 patients a revision to reverse total shoulder replacement was performed.

Fracture morphology was not seen to have any correlation with overall complication rate in either the LBN or PHILOS groups (*p* = 0.177 and *p* = 0.583).

## Discussion

At short-term follow-up LBN osteosynthesis yielded similar outcomes and complication rates to PHILOS plate fracture fixation in an elderly patient population. However a significantly higher rate of secondary loss of reduction with concomitant screw penetration was seen in the PHILOS cohort, confirming the hypothesis of this study.

With a median age and gender adjusted Constant Score of 94%, a DASH score of 41 points, a VAS of 0 and an active range of motion of 127° of flexion and 122° of abduction, both groups in the present study demonstrated positive clinical outcomes at 12-month follow-up. These objective measures are supported by the patients’ subjective perception that they had achieved “good” shoulder function by that point. Except for improved DASH score in the LBN cohort at final follow-up, no significant differences in functional outcome was observed between groups at any follow-up time-point. Likewise, no difference in mean duration of surgery, mean intraoperative image intensifier time or postoperative hospital stay, was seen between the two groups.

Recently Gracitelli et al. [4] published a prospective, randomised control study, also including more complex three-part PHFs, comparing the Centronail (Orthofix, Verona, Italy) with the PHILOS plate. Four-part PHFs were, however, excluded. At 12 months follow-up mean Constant score was 70.3 in the nail group (*n* = 32) and 71.5 in the plate group (*n* = 33), not dissimilar from the present study. Recorded DASH scores, however, demonstrated less residual disability at 12-months, with a mean of 18.1 and 14.3 points for nail and plate respectively, but with higher persisting pain VAS of 1.7 and 1.3. These observed differences may be due to the significant lower mean age at surgery of 64.5 and 66.4 years in that study. Similarly to the present study, clinical outcome scores were seen to improve between 3, 6 and 12 months postoperatively, without significant differences between cohorts.

The other study on this topic with a prospective randomized controlled design was published by Zhu et al. in 2011 [16]. The authors prospectively followed patients treated with a Proximal Humeral Nail (PHN, Synthes, DePuy Synthes, Solothurn, Switzerland) and patients treated with a locking proximal humeral plate system (LPHP or PHILOS, DePuy Synthes, Solothurn, Switzerland) over a 3-year follow-up period. At 12 month follow-up their patients exhibited improved Constant score (88.0 PHN and 92.0 LPHP/PHILOS) and active forward elevation (151° PHN and 155° LPHP/PHILOS) as compared to the present study. VAS scores were comparable (1.0 PHN and 0.5 LPHP/PHILOS). Zhu et al., however, only included simple two-part surgical neck PHFs and in a considerably younger patient population (54.8 years PHN and 50.5 LPHP/PHILOS at the time of surgery) than the present study (71.1 years LBN and 77.1 years PHILOS).

Outcomes following three-part PHF osteosynthesis in a large multi-centre study, comparing the same implants as Zhu and colleagues, were published by Konrad et al. in 2012 [22]. With a mean age and gender adjusted Constant score of 89% in the nail group and 87% in the plate group, outcomes were comparable to the present study’s results, though again with mean patient age of 64.8 in the nail and 65.4 in the plate group, in a somewhat younger cohort. In contrast to the present findings, however, the average duration of surgery was significantly shorter in the nailing group but with a significantly longer image-intensifier exposure time.

Gradl et al. [20] performed a retrospective match-pair analysis in a large patient population (*n* = 152), comparing the outcomes of a Locking Proximal Humerus Plate (Mathys, Bettlach, Switzerland) with the Targon PH nail (Braun-Aesculap, Tuttlingen, Germany) across a wide variety of PHF patterns, including fracture dislocation. Age and gender adjusted Constant scores were seen to improve consistently over the post-operative follow-up period, again with no significant inter-group difference at any follow-up point and for all fracture patterns. With an adjusted Constant score of 80% in the nail group and 77% in the plate group at final, 12-month, follow-up, their clinical outcomes were considerably poorer than those of the present study (90% LBN and 95% PHILOS). Additionally, Gradl and colleagues observed poorer clinical outcome scores in the nail group for four-part PHFs as compared to two- and three-part, and a higher overall rate of significant complications requiring re-operation within the Targon nail group.

A higher incidence of failure of fixation with nail osteosynthesis of four-part PHFs, as compared to more simple fracture patterns, was also identified by Sosef et al. [19] in 20 consecutive patients treated with a T2 nail (Stryker, Duisburg, Germany), where four-part PHFs were generally seen to achieve poorer outcomes. At a mean 19 months follow-up patients showed an overall age and gender adjusted Constant score of 62%, as compared to 90% at 12-months in the present study.

In contrast to the findings of Gradl et al. [20] and Sosef et al. [19], more complex fracture morphology did not appear to affect final clinical outcome, or complication rate in the present study. This seems likely to be due to the modern design of the LBN, with its four cross-locking cancellous screws with broad washers, and the locking blade providing a more stable fracture fixation as compared to the previous designs of proximal humeral nailing systems tested, making it a more suitable implant for the treatment of complex osteoporotic fractures.

Furthermore, in the current study secondary varus dislocation with screw cut-out was found to be a characteristic complication in the PHILOS group. This is again in contrast to the results of Gradl et al. [20] who found glenohumeral cut-out to be a characteristic issue with the Targon PH nail (13% nail and 7.8% plate). This is perhaps all the more significant given the younger patient cohort (63 years), and therefore superior bone quality, in that study as compared to the present cohort (75.6 years). Again this seems likely to be due to the LBN nail design, and particularly its locking blade, providing superior medial calcar support and so superior outcomes as compared to those previously published for alternative nailing systems without calcar support or those of the PHILOS plate, with its calcar support screws, observed in the present study.

This hypothesis is also supported by recent biomechanical studies by Rothstock et al. [33] and Wanzl et al. [34]. Both authors tested the MultiLoc nail (DePuy Synthes, Solothurn, Switzerland), a nailing system which similarly to the LBN provides an additional calcar support screw and proximal screw-in-screw options, against nailing systems without these features and found a positive impact on the failure load. However, while the concept of calcar support in proximal humerus plating is generally accepted, the role of calcar support devices in proximal humerus nailing currently remains an unanswered question. Further clinical studies are needed to clarify this point [10, 13, 14, 35–38].

Besides loss of reduction, reported complications in the present study include secondary greater tuberosity resorption, humeral head migration, humeral head osteonecrosis, axillary nerve lesion, adhesive capsulitis as well as implant malposition not affecting shoulder function.

Overall, 29 complications were seen in 23 patients, and 14 patients, with 16 complications, subsequently required re-operation. With 16 complications in 11 patients in the PHILOS group (*n* = 32), as compared to 13 complications in 12 patients in the LBN group (*n* = 36), plating appeared to demonstrate a higher incidence of complications, though this was not found to be statistically significant. The same pattern was observed for re-operation rates, where 5 revisions were indicated in the LBN group and 11 in the PHILOS group, again not reflecting a statistically significant difference in this study cohort. Secondary varus collapse with screw cut-out was the most commonly observed complication in the overall study population and, as described previously, was the only complication that demonstrated a statistically significant inter-group difference.

This tendency towards higher complication rates in proximal humerus plating is consistent with the results described by Zhu et al. [16], Konrad et al. [22] and Hardeman et al. [3], but in contrast to the findings of Gracitelli et al. [4] who saw a trend of increased complications in proximal humerus nailing.

The overall rate of complications and re-operation in the current study appears high at first glance, however, the clinical significance of these complications must also be considered, particularly in the light of the inclusion of subjective suboptimal implant positioning, without clinical consequence, in this analysis. Likewise, consideration must also be taken of the impact of the elderly patient cohort, with their associated comorbidities and increased risk of perioperative complications. Furthermore, the postoperative treatment in this unit, with no active range of motion restriction, no immobilization and limited weight bearing for 6 weeks when possible, is much more aggressive than other reports in the literature [16]. The principal aim in geriatric fracture care in this unit is to maintain patient autonomy in daily life and to avoid permanent care dependency. This often means that patients are obliged to load the operated shoulder in the immediate post-operative period, particularly if dependent on walking aids, so putting the fracture fixation at further risk.

In summary, many factors that may be fracture associated, patient associated or implant associated influence the outcomes of PHF fixation, making a direct comparison between studies difficult. Overall, the clinical results presented here appear equivalent to, or even an improvement on those in the published literature, despite an elderly population with complex and frequently osteoporosis associated PHFs.

The principal findings and trends of the current study are also consistent with the results of a recent meta-analysis on this topic. Sun et al. [39] performed a systematic review of 13 comparative studies and included 958 patients. The authors reported similar results for Constant & DASH scores, VAS, forward elevation and total complication rates in both groups. In the pooled data, screw penetration occurred in 13.1% of patients in the looking plate group and in 6.2% in the intramedullary nail group, representing a significant difference.

There are several limitations in the present study that must be considered. Firstly, with a follow-up of 12 months, long-term complications may be underestimated, however previous studies on PHFs in an elderly population have reported minimal changes of clinical outcomes between 12 and 24 months postoperatively [40, 41]. Furthermore, a study in an elderly population inevitably has a high drop-out rate due to immobility, comorbidities or death of the patients, presenting difficulties for longer term follow-up.

Secondly, this study only recruited a limited study population of 68 patients, which limits the power of the statistical analysis, increasing the risk of type II error.

Finally, though osteoporosis is a primary risk factor for failure of PHF fixation, bone mineral density was not systematically evaluated in this patient cohort, although prevalence may be assumed to be high.

These limitations are, however, mitigated by a number of strengths, including a homogeneous elderly population, adherence to strict inclusion and exclusion criteria, a prospective and randomized design, independent observation, high level surgeon experience and the use of two defined implants.

## Conclusions

At short term follow-up LBN osteosynthesis yielded similar outcomes and complication rates as compared to PHILOS plate fracture fixation in an elderly patient population. The overall complication rate and rate of re-operation is high. Secondary loss of reduction with concomitant screw penetration was found to be a characteristic complication in PHILOS plating. PHFs in an elderly patient population remain a challenging situation for orthopaedic trauma surgeons but, with appropriate surgeon experience, a locking blade nail appears to be a safe and effective treatment modality. In our practice, plate osteosynthesis, however, still remains a viable alternative for the treatment of PHFs in elderly patients in cases where fracture morphology does not allow nailing (i.e. head-splitting fractures).

## Abbreviations

AO: Arbeitsgemeinschaft für Osteosynthesefragen
DASH: Disabilities of the Arm, Shoulder and Hand
LBN: locking blade nail
LPHP: locking proximal humeral plate system
PHF: Proximal humeral fractures
PHILOS: Proximal Humerus InterLocking System
VAS: Visual Analog Scale
